# GKN2 promotes oxidative stress-induced gastric cancer cell apoptosis via the Hsc70 pathway

**DOI:** 10.1186/s13046-019-1336-3

**Published:** 2019-08-05

**Authors:** Ziqiang Zhang, Hongyuan Xue, Yuanqiang Dong, Jun Zhang, Yida Pan, Liubin Shi, Panpan Xiong, Jie Zhu, Wenshuai Li, Wanwei Zheng, Jie Liu, Jianjun Du

**Affiliations:** 1 Departments of General Surgery, Huashan Hospital, Fudan University, 12 Middle Urumqi Road, Shanghai, 200040 People’s Republic of China; 2 Departments of Digestive Diseases, Huashan Hospital, Fudan University, 12 Middle Urumqi Road, Shanghai, 200040 People’s Republic of China; 3 Departments of Digestive Diseases, Dongfang Hospital, Tongji University, Shanghai, China

**Keywords:** GKN2, Oxidative stress, Gastric cancer, Apoptosis

## Abstract

**Background:**

The GKN2 is a secretory protein, whose levels decrease in gastric cancer. The present study aimed to investigate the expression, function and mechanism of action of GKN2 in gastric cancer.

**Methods:**

Molecular biology assays were performed to elucidate the function and underlying mechanisms of GKN2 in gastric cancer under stress-induced condition in vivo and in vitro. Clinical specimens were used to assess the correlation of GKN2 and prognosis.

**Results:**

We found that overexpression of GKN2 significantly enhanced apoptosis and growth arrest in vitro. GKN2 expression increased in gastric cancer cells exposed to hydrogen peroxide and promoted reactive oxygen species-induced mitochondrial dysfunction and resulted in increased cell apoptosis via inhibition of NF-κB signaling pathway and activation of JNK signaling pathway through the direct interaction of GKN2 with Hsc70. Trefoil factor 1 might contribute to the tumor suppressing effects of GKN2. MiR-216a downregulated *GKN2* expression. GKN2 also inhibited xenograft tumor growth and was an independent and significant prognostic factor for patients with gastric cancer treated with oxaliplatin.

**Conclusions:**

Taken together, our data indicate that GKN2 may increase sensitivity of GC cells to the drugs which increase ROS levels in tumors. Inhibition of the interaction between GKN2 and Hsc70 could attenuate the effects induced by GKN2. GKN2 overexpression could be used to determine the subgroup of patients to obtain the more favorable outcome of oxaliplatin treatment and may be used as biomarker of the prognosis of this cancer.

**Electronic supplementary material:**

The online version of this article (10.1186/s13046-019-1336-3) contains supplementary material, which is available to authorized users.

## Background

Gastric cancer (GC) is one of the most common malignancies worldwide [1] and the second biggest health burden in China [2]. The pathogenic mechanisms of GC have been characterized as multifactorial and complex, but the specific set of factors that promote GC development and progression and their dynamic interactions remains poorly understood.

Oxidative stress, an imbalance between oxidant production and antioxidant activity, refers to the pathophysiology of a wide range of acute and chronic conditions [3]. Damage from oxidative stress is involved in many types of diseases, including neurological diseases, diabetes, atherosclerosis, arthritis, inflammation, and several types of cancer, including GC [4].

The tumor microenvironment is characterized by different stress conditions such as hypoxia and nutrient deprivation induced by defective tumor vasculature, or genotoxic and oxidative stress caused by rapid cell division or therapy [5–7]. Tumor cells need to manage these stresses, and tumor progression and ultimately patient outcome will be determined by the resistance of tumor cells to the different stresses they are exposed to. At the cellular level, the stress response depends on both the tumor energy status and its adaptive response, which contribute to the maintenance of cell survival. However, stress adaptation happens at the expenses of tumor proliferation; therefore, tumor cells need to develop strategies to survive stress and proliferate.

MicroRNAs (miRNA) participate in many normal biologic processes, including cell proliferation, differentiation, apoptosis, and organ development. Most miRNAs function by negatively regulating gene expression by directly binding to the 3′-untranslated region (3′-UTR) of a target gene mRNA, which induces mRNA cleavage or translational repression [8]. Mir-216a plays an important role in various kinds of cancers [9, 10]. At the same time it also functions as a modulator in response to oxidative stress [11].

The gastrokine 2 (*GKN2*), also known as gastric dramatic down-related gene (*GDDR*), was discovered and cloned for the first time by our team [12]. GKN2, a small (~ 18 kDa) protein belonging to the BRICHOS protein superfamily, is characterized by an approximately 100 amino acid BRICHOS domain, and plays a role in inflammatory diseases, dementia, and cancer [13, 14]. While all BRICHOS proteins are secreted or processed to generate mature peptides, GKN2 is unique in being almost exclusively expressed within, and secreted by, the mucus-producing epithelial cells of the stomach [12, 13, 15]. GKN2 is evolutionary conserved in mammals and higher vertebrates [13, 16, 17].

While GKN2 is abundantly expressed in surface mucus cells of the normal human stomach, its levels decrease in gastric adenocarcinoma as well as in tumor cell lines [12]. Conversely, GKN2 has been identified as the most upregulated gene in an expression microarray assay in the gastric transcriptome after eradication of *Helicobacter pylori* and cure of mucosal inflammation [18]. These observations suggest that GKNs, and, specifically, GKN2, play a vital role in the homeostatic regulation of mucosal immunity and/or in stomach-specific tumor-suppression. The mechanism of action of GKNs is still obscure, as well as their cognate receptors and the signaling pathways they regulate [13]. Interestingly, some studies on the GKN2/trefoil factor (TFF)1 heterodimer have suggested that GKN2 might have homeostatic and/or tumor-suppressor activities via TFFs [19, 20].

To identify the impact of GKN2 loss in the context of stress, we analyzed the expression of GKN2 in GC cells exposed to hydrogen peroxide (H_2_O_2_). Additionally, we investigated GKN2 effects on cell viability, proliferation and apoptosis under stress conditions. This study suggests that GKN2 might affect the sensitivity of GC cells to oxidative stress. Loss of GKN2 results in resistance of cells to oxidative stress, which can justify the tumor suppressor function of GKN2.

## Methods

### Cell culture

MGC-803 (MGC), SGC-7901 (SGC) and 293 T cells were obtained from the Cell Bank of Chinese Academy of Medical Science (Shanghai, China). GC cell lines were cultured in Roswell Park Memorial Institute-1640 containing 10% fetal bovine serum (Invitrogen Life Technology, Carlsbad, CA, USA), penicillin (100 U/ml), and streptomycin (100 mg/ml). 293 T cells were cultured in Dulbecco’s modified Eagle’s medium containing 10% fetal bovine serum, penicillin (100 U/ml), and streptomycin (100 mg/ml). H_2_O_2_ was purchased from Sangon Biotech (Shanghai, China).

### Cell transfection and overexpression

Cells were transfected with small interfering RNA (siRNA) or plasmid vectors using Lipofectamine2000 (Invitrogen Life Technology) according to the manufacture’s instruction. The sequences of siRNAs were as follows: siHsc70–1: 5′-GCUGGUCUCAAUGUACUUATTUAAGUACAUUGAGACCAGCTT-3′; siHsc70–2: 5′-GGCCAGUAUUGAGAUCGAUTTAUCGAUCUCAAUACUGGCCTT-3′; siTFF1–1: 5′-AGACAGAAUUGUGGUUUUCTT-3′; siTFF1–2: 5′-AUGGUAUUAGGAUAGAAGCACCAGG-3′. The siRNAs were from GenePharma (Shanghai, China). The pcDNA3 plasmid, pcDNA3-Hsc70 plasmid, pcDNA3-GKN2 plasmid, pcDNA3-GKN2 mutation plasmid and HA labeled ubiquitin enzyme (Ub-HA) plasmid were purchased from Fubio Biological technology (Suzhou, China). The mimics and inhibitors of miR-216a were purchased from Biotend (Shanghai, China).

### Cell proliferation and clonogenic assays

Cells (1 000 cells/well) were seeded into 96-well plates for a cell counting kit-8 (CCK8) colorimetric assay (Dijindo, Japan) according to the manufacture’s specifications. For the clonogenic assay, the cells were seeded into 6-cm plates and cultured for 14 days. The colonies on the plates were fixed with 4% paraformaldehyde, stained with crystal violet and counted.

### Western blotting

Cell lysates were extracted with a cell lysis buffer (Beyotime, Hangzhou, China) and the protein concentration was quantified using an Enhanced BCA Protein Assay Kit (Beyotime). The primary antibodies used were as follows: anti-p65 (1:1000), anti- phosphorylated p65 (1:1000), anti-JNK (1:1000), anti-phosphorylated JNK (1:1000), anti-glyceraldehyde 3-phosphate dehydrogenase (GAPDH) (1:1000), anti-cleaved caspase-3 (1:1000), anti-cleaved caspase-9 (1:1000), anti-cleaved PARP (1:1000) (CST, Danvers, MA, USA); anti-GKN2 (1:1000), anti-Hsc70 (1:1000) (Abcam, Cambridge, MA, USA). Anti-rabbit antibody (1:2000) and anti-mouse antibody antibodies (1:2000) (CST) were used as secondary antibodies. Western blot was performed as previously described [21].

### Quantitative real-time polymerase chain reaction (qRT-PCR)

QRT-PCR assays were conducted on a Bio-Rad quantitative PCR system (Hercules, CA, USA). For data analysis, raw counts of the target genes were normalized to those of the house keeping gene averaged for the same time point and condition. Counts are reported as fold change relative to the untreated control. All primers were designed and synthesized by Genewiz (Suzhou, China). The following primers were used: GKN2-F, 5′-AGAGCCTGCTTTATCCTGAAGA-3′; GKN2-R, 5′-ACTTGACCCAGGTGTATTTGC-3′. GAPDH-F, 5′-CTCACCGGATGCACCAATGTT-3′; GAPDH-R, 5′-CGCGTTGCTCACAATGTTCAT-3′. The miRcute Plus miRNA First-Strand cDNA Synthesis Kit was used for miRNA reversely transcription (TIANGEN BIOTECH CO., Beijing, China).

### Luciferase assays

Using genomic DNA from 293 T cells as the template, the DNA sequence of the GKN2 3′-UTRs containing the potential miR-216a binding site was amplified and cloned into the XbaI site immediately downstream of the stop codon in the pGL3-promoter vector (Promega, Madison, WI, USA). Using four overlapping primers, the predicted miR-216a binding site was then replaced by a mutated 18 bp-long fragment generating a pGL3 reporter plasmid with the mutated GKN2 3′-UTR. 293 T cells were seeded in 96-well plates and co-transfected with 5 ng of pRL-renilla (Promega; internal control), 50 ng of the 3′-UTR pGL3-promoter reporter and 150 ng of pSilencer-miR-126a (or pSilencer4.1CMV-negative). Forty-eight hours after transfection, the firefly and Renilla luciferase activities were assayed using the Dual-Glo Luciferase assay system (Promega). All experiments were performed in triplicate and repeated at least three times.

### Immunofluorescence and immunohistochemistry

Cells were seeded in 6-well plates, incubated for 24 h and treated with H_2_O_2_ or phosphate-buffered saline (PBS) for 6 h. The primary antibodies used were anti-GKN2 and anti-Hsc70 (Abcam). 4′,6-diamidino-2-phenylindole (DAPI; Solarbio, China) was used for nuclear staining. Alexa Fluor® 488 anti-rabbit and Alexa Fluor® 594 anti-mouse antibodies (CST) were used as secondary antibodies. Immunofluorescence assays were performed as previously described [22]. Tissue samples were fixed in 4% paraformaldehyde and embedded in paraffin. Anti-GKN2 (1:200; Abcam) and anti-Ki67 (1:200; CST) were used as primary antibodies. Immunohistochemistry was performed as previously described [23]. GC tissues and adjacent non-malignant gastric tissues were obtained from the Huashan Hospital (Shanghai, China). The use of all tissue samples was approved by the Institutional Ethics Review Board of the Huashan Hospital.

### Superoxide dismutase activity

After exposure to H_2_O_2_ treatment, superoxide dismutase activity of cells was detected by Total Superoxide Dismutase Assay Kit (Beyotime) according to the manufacturer’s instruction.

### Flow cytometric analysis

Flow cytometry was performed to analyze the cell cycle, apoptotic cells, mitochondrial membrane potential and reactive oxygen species (ROS) production. Dead Cell Apoptosis Kit (Invitrogen Life Technology) was used to determine cell apoptosis. The cells were analyzed for apoptosis immediately at the end of H_2_O_2_ treatment. MitoProbe™ JC-1 Assay Kit (Invitrogen Life Technology) was used to detect mitochondrial membrane potential. Tali™ Cell Cycle Kit (Invitrogen Life Technology) was used to identify cell cycle. Cells were pretreated with H_2_O_2_ then cultured for another 24 h before cell cycle evaluation. ROS production was analyzed using the DCFH-DA probe (Beyotime). After exposure to various experimental conditions, flow cytometry was performed according to the manufacturer’s instruction.

### Immunoprecipitation and sequential mass spectrometry (MS)

Cells were lysed with a cell lysis buffer (CST) supplemented with complete protease inhibitor cocktail (Roche, Switzerland). Protein-G-Agarose beads (Invitrogen Life Technology) were incubated with anti-myc antibody or non-specific rabbit IgG antibody overnight. Protein lysates were added to the beads and incubated overnight. After SDS-PAGE, gel fixation, Coomassie staining (Merck, Kenilworth, NJ, USA), and mass spectrometry were carried out by PoolingMed Co., Ltd. (Hangzhou, China).

### Phospho-specific protein microarray analysis

The Phospho Explorer Antibody Microarray was conducted by Full Moon BioSystems Inc. (Sunnyvale, CA, USA). Whole-cell lysates from cell cultures treated with H_2_O_2_ (300 μM) were harvested 6 h after treatment using a Protein Extraction Buffer (Full Moon BioSystems Inc.) and transferred to Full Moon BioSystems Inc., on dry ice. The array consists of 1318 phospho-specific antibodies. In brief, proteins were labeled with biotin and placed on pre-blocked microarray slides. After washing, the detection of total and phosphorylated proteins was conducted using Cy3-conjugated streptavidin. The expression of phosphorylated proteins was normalized to the corresponding total protein expression. Fold change was calculated as follows: phosphorylation of H_2_O_2_-treated cells/ phosphorylation of untreated cells. This experiment was carried out once. Where indicated, protein phosphorylation data were confirmed by western blotting.

### Tumor xenograft mouse models

The animal care and experimental protocols were approved by the Animal Ethics Committee of Shanghai Medical College, Fudan University, China. Male BALB/c nude mice (4-weeks old) were obtained from Shanghai Research Center of the Southern model organisms. Mice were kept in specific pathogen-free conditions. The mice were randomly divided into groups before injection. Cells (10^7^) were suspended in 200 μl PBS and subcutaneously injected in mice. Mice were treated with PL at the dose of 4 mg/kg body weight by intraperitoneal injection once every other day. Four weeks post-injection, the mice were sacrificed and the tumors were isolated and weighed.

### Databases and statistics

We computationally screened microRNA targeting GKN2 using the miRWalk database (http://mirwalk.umm.uni-heidelberg.de/). Results are expressed as the mean ± standard deviation (SD). The Student’s t-test and one-way ANOVA were used to determine the significance of differences between groups. A *P*-value of < 0.05 was considered significant and a P-value of < 0.01 was considered very significant. Overall survival rates were calculated by the Kaplan–Meier method and differences were analyzed by the log-rank test. Univariate and multivariate analyses were performed using the Cox proportional hazards regression model. All statistical analyses were performed using the GraphPad Prism (GraphPad Software Inc., San Diego, CA, USA).

## Results

### Oxidative stress increase the expression of GKN2 in GC cell lines

**Fig. 1:**
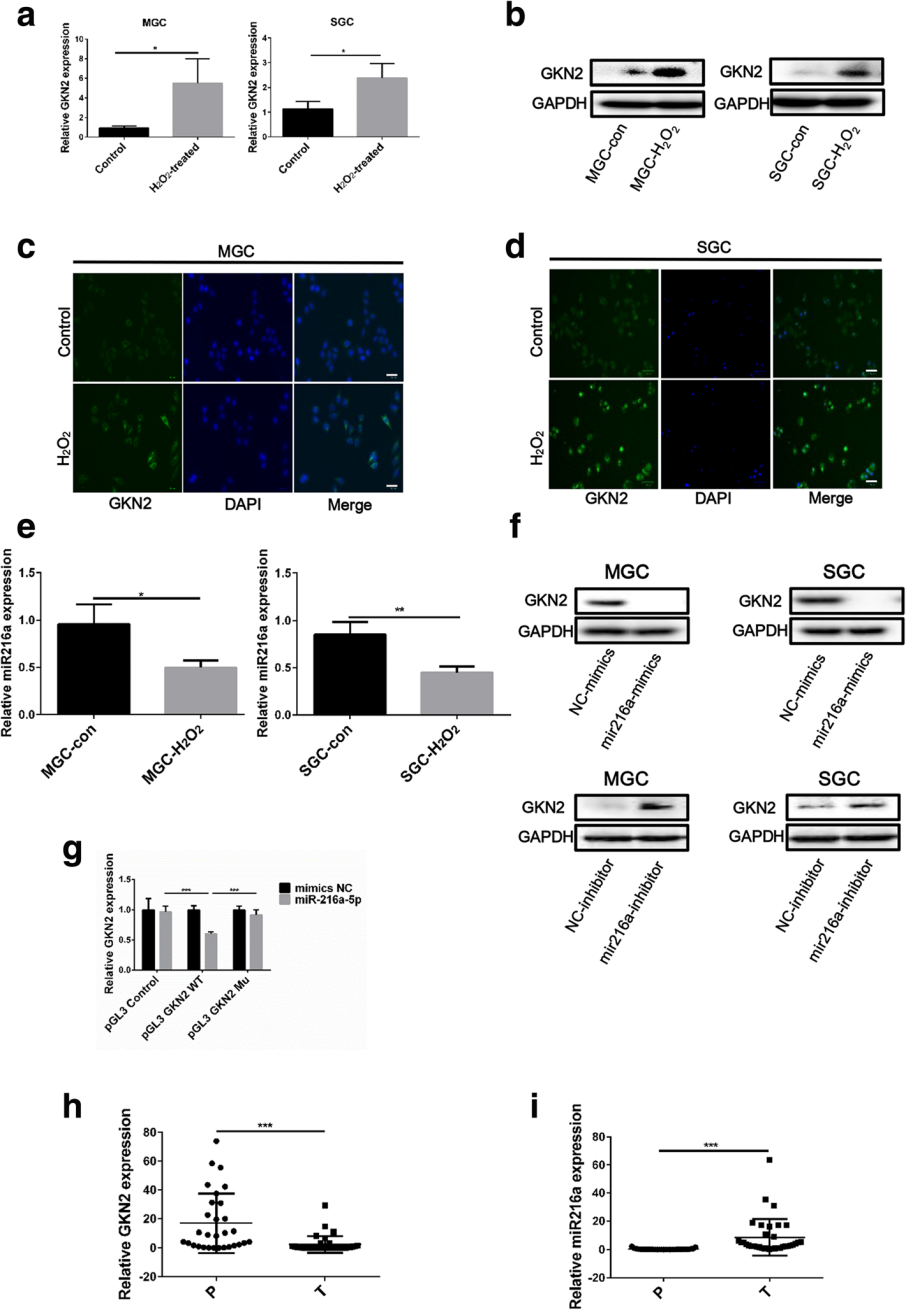
Stress-induced GKN2 expression in GC cell lines. **a**QRT-PCR detection of GKN2 mRNA expression in MGC and SGC cells after H_2_O_2_ (300 uM) treatment for 6 h. **b** GKN2 expression detected by western blotting after H_2_O_2_ (300 uM) treatment for 6 h. **c**-**d** Immunofluorescence detection of GKN2 expression after H_2_O_2_ (300 uM, 6 h) treatment. MGC and SGC cells were pre-treated with or without H_2_O_2_ (300 uM) for 6 h. Scale bar: 50 μm. **e** QRT-PCR detected miR-216a expression after H_2_O_2_ (300 uM, 6 h) treatment. **f** Western blot detection of GKN2 expression in MGC and SGC cells transfected with miR-216a mimics (50 nM) and inhibitor (50 nM) for 48 h. (g) MiR-216a significantly decreased luciferase report gene activities of GKN2 3’-UTR pGL3-promoter. **h**-**i** GKN2 and miR-216a expression was detected by qRT-PCR in gastric tumor tissue and peripheral tissue. P: para-carcinoma tissues; T: tumor tissues. Dots are the values of GKN2 and mir-216a expression; the error bar indicates the SD. Asterisks indicate statistically significant differences from each other; *p < 0.05, **p < 0.01, ***p < 0.001

To investigate whether stress could affect GKN2 expression, we examined the effects of H_2_O_2_ on GC cell lines (MGC and SGC). The observed enhancement of GKN2 expression in GC cell lines indicated that stress conditions induced GKN2 mRNA and protein expression (Fig. 1a, b). Immunofluorescence analysis also showed increased levels of GKN2 in GC cell lines with H_2_O_2_ treatment; in contrast, control cells, not treated with H_2_O_2_, had lower levels of GKN2 in the cytoplasm (Fig. 1c, d). Interestingly, we found H_2_O_2_ downregulated the expression of miR-216a (Fig. 1e), which was identified as a possible microRNA targeting *GKN2* through a computational screen using an open access software. The mimics or inhibitors of miR-216a were transfected into GC cell lines and the expression of GKN2 was detected (Fig. 1f). Next, we investigated whether the expression of GKN2 was regulated by miR-216a in luciferase assays. We constructed luciferase reporter plasmids (in the pGL3 vector) with the luciferase coding region followed by the wild type or mutated 3′-untranslated region (UTR) of *GKN2* and found that the luciferase activity of the plasmid with the wild type *GKN2* 3′-UTRs was significantly inhibited by miR-216a, while the luciferase activity of the reporter with the mutated GKN2 3′-UTR was not (Fig. 1g). These data suggested that miR-216a targets *GKN2*. We then tested the expression of GKN2 and miR-216a in GC and their adjacent tissues and found that GKN2 was downregulated in GC tissues; inversely, miR-216a was upregulated (Fig. 1h, i; Additional file 2: Table S1).

### GC cell lines overexpressing GKN2 are hypersensitive to oxidative stress

**Fig. 2:**
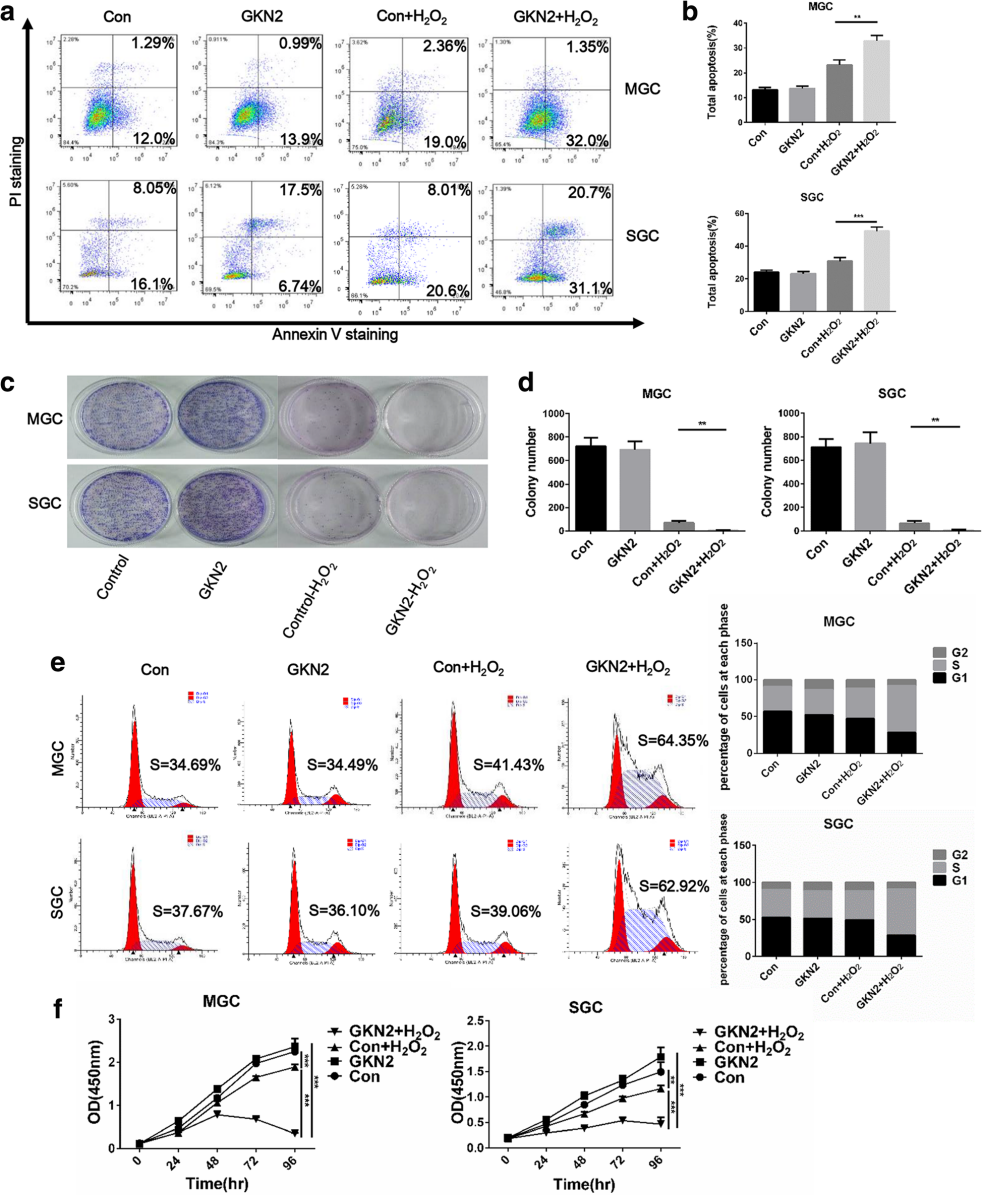
The stress-induced pro-apoptotic effect of GKN2 on GC cell lines. **a**-**b** Analyses of apoptosis with or without H_2_O_2_ (300 uM) concentration for 6 h in GC cell lines. **c**-**d** Comparison of colony formation in MGC and SGC cells with or without H_2_O_2_ (300 uM) pre-treatment for 6 h. **e** Analyses of cell cycle after PI staining in GC cell lines after H_2_O_2_ (300 uM) treatment for 6 h. Data are presented as the mean of three independent experiments. **f** Comparison of proliferation in MGC and SGC cells with or without H_2_O_2_ (300 uM) pre-treatment for 6 h. GKN2 overexpressing MGC cells (IC50 528.4 ± 29.5 μM) and SGC cells (IC50 539.2 ± 31.6 μM) were significantly more sensitive to H_2_O_2_ compared to control MGC cells (IC50 660.4 ± 29.4 μM, *P* < 0.01) and SGC cells (IC50 663.0 ± 25.6 μM, *P* < 0.01), respectively. Data are presented as mean ± SD from three independent experiments with each running in triplicate. (*n* = 3, **p < 0.01, ***p < 0.001)

To address the role of GKN2 on GC cells, we first overexpressed GKN2 in MGC and SGC cells using lentiviral vectors. Each GC cell line infected with GKN2 lentiviral vectors showed efficient overexpression of GKN2, as determined by qRT-PCR and western blot (Additional file 1: Figure S1a, b). We observed the effects of GKN2 on apoptosis of MGC and SGC cells and found that overexpression of GKN2 promoted apoptosis induced by oxidative stress (Fig. 2a, b). We also performed clonogenic assays and found that GKN2 overexpression notably suppressed clonogenic survival by inhibiting colony formation under oxidative stress, as shown in Fig. 2c-d. Cell cycle assays showed S-phase arrest in MGC and SGC cells after treatment with H_2_O_2_ and GKN2 overexpression (Fig. 2e). In addition, we found that GKN2 overexpression significantly decreased the viability of cells under oxidative stress (Fig. 2f). Taken together, our results demonstrated that GKN2 inhibited cell proliferation and promoted apoptosis in GC cells.

### Knockdown of GKN2 promotes the growth of GC cell lines subjected to oxidative stress

To further assess the role of GKN2 in the proliferation and apoptosis of GC cells, the CRISPR/Cas9 system was used to knock out *GKN2* in MGC and SGC cells. QRT-PCR and western blot assays were performed to confirm silencing of *GKN2* at the mRNA and protein level (Additional file 1: Figure S1c, d). We found that silencing of *GKN2* inhibited the apoptosis of cells after H_2_O_2_ treatment (Additional file 1: Figure S2a, b). Moreover, colony formation assays showed that silencing of *GKN2* notably enhanced the clonogenic survival of the cells (Additional file 1: Figure S2c, d). In addition, knockout of *GKN2* reduced the percentage of cells in S-phase arrest (Additional file 1: Figure S2e), and enhanced proliferation in both MGC and SGC cells with H_2_O_2_ treatment (Additional file 1: Figure S2f). These data further supported the inhibitory role of *GKN2* in the regulation of cell proliferation and survival.

### GKN2 promotes ROS-induced mitochondrial dysfunction

We next examined whether the effect of H_2_O_2_ on GC cells was due to increased ROS levels, as it happens in other cancer types [24]. After treatment with H_2_O_2_, superoxide dismutase activity was significantly lower in GC cell lines overexpressing GKN2 (Additional file 1: Figure S3a). ROS levels in MGC and SGC cells were examined by flow cytometry using the redox-sensitive fluorescent probe 2’-,7’-dichlorofluorescein diacetate (DCFH-DA). Exposure to H_2_O_2_ for 6 h led to an increase of ROS levels in the cells and pre-treatment with glutathione (GSH) reversed this effect, suggesting that GKN2 plays a role in stress-induced apoptosis in cancer cells (Additional file 1: Figure S3b). Mitochondria are important in the regulation of apoptosis. Loss of mitochondrial membrane potential (△ψm) is harmful for cells and results in the release of cytochrome C into the cytosol [25] and apoptosis. Therefore, we analyzed whether H_2_O_2_-induced apoptosis was linked to mitochondrial homeostasis by flow cytometry upon staining with the mitochondrial membrane potential probe JC-1. We found that GKN2 contributed to loss of mitochondrial membrane potential after exposure to H_2_O_2_ for indicated time, while pre-treatment with GSH abolished this effect (Additional file 1: Figure S3c, d).

### GKN2 promotes apoptosis via the caspase pathway

**Fig. 3:**
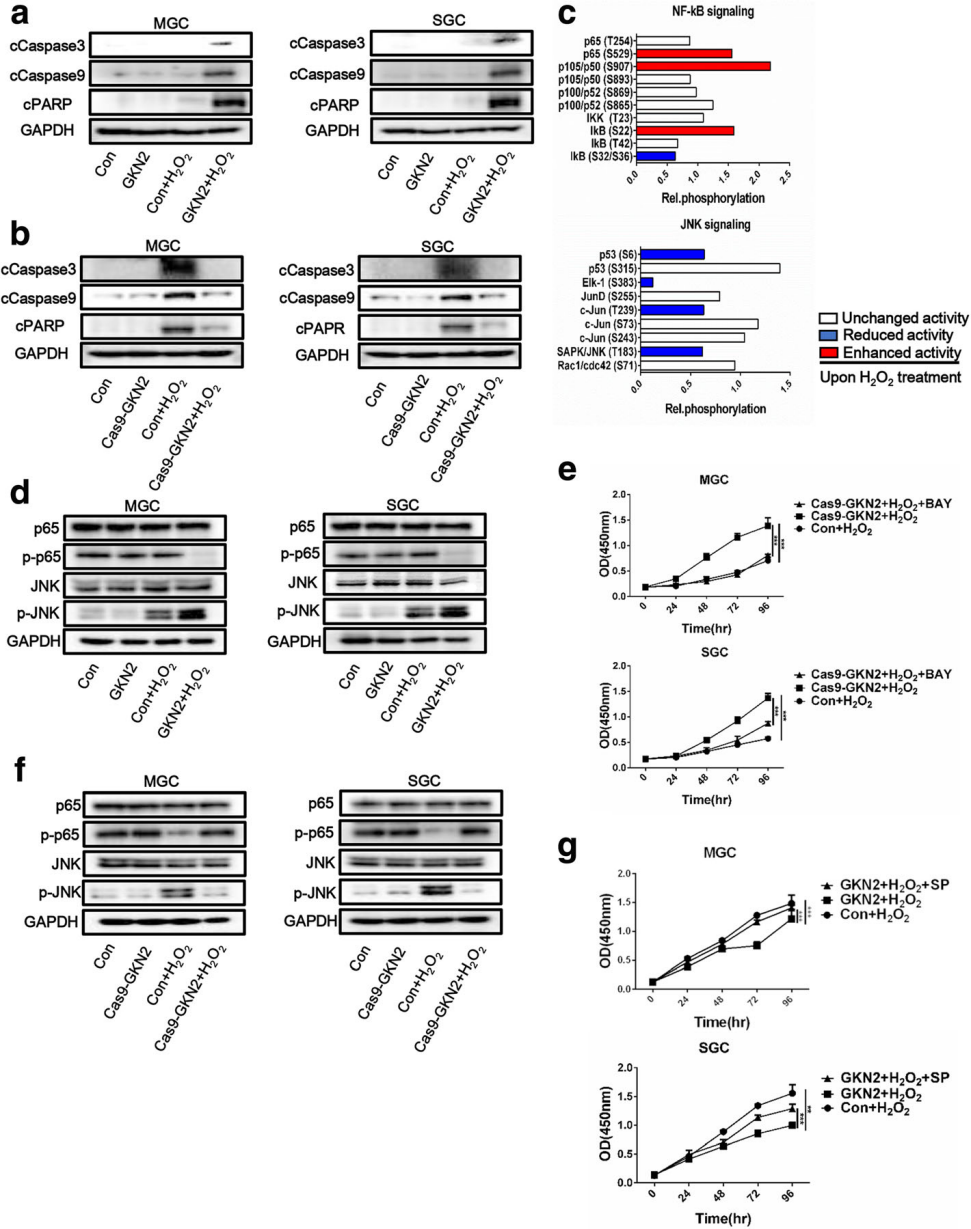
Effects of GKN2 on NF-κB and JNK signaling. **a**-**b** Western blotting detection of pro-apoptotic protein in GC cell lines after H_2_O_2_ (300 uM) treatment for 6 h. **c** The Phospho Explorer Antibody Array was used to screen total cell lysates from untreated and H_2_O_2_-treated cell cultures. Data show fold change of indicated phosphoproteins upon H_2_O_2_ (300 uM) treatment for 6 h after normalization to total protein expression. **d** & **f** Western blotting detection of the protein expression and phosphorylation in GC cell lines after H_2_O_2_ (300 uM) treatment for 6 h. (**e**) Comparison of the proliferation of GC cell lines after H_2_O_2_ (300 uM) treatment for 6 h with or without inhibition of NF-κB by 10 uM bay11–7082. Cells were pretreated with the inhibitors for 2 h and maintained in culture. GKN2 silencing MGC cells (IC50 676.7 ± 27.9 μM) and SGC cells (IC50 644.8 ± 30.0 μM) with inhibition of NF-κB were significantly more sensitive to H_2_O_2_ compared to GKN2 silencing MGC cells (IC50 807.4 ± 45.4 μM, *P* < 0.05) and SGC cells (IC50 836.8 ± 30.5 μM, *P* < 0.01), respectively. (**g**) Comparison of the proliferation of GC cell lines after H_2_O_2_ (300 uM) treatment for 6 h with or without inhibition of JNK by 10 uM SP600125. Cells were pretreated with the inhibitors for 2 h and maintained in culture. GKN2 overexpressing MGC cells (IC50 603.4 ± 23.2 μM) and SGC cells (IC50 611.3 ± 25.5 μM) with inhibition of JNK were significantly less sensitive to H_2_O_2_ compared to GKN2 overexpressing MGC cells (IC50 528.4 ± 29.5 μM, *P* < 0.05) and SGC cells (IC50 539.2 ± 31.6 μM, *P* < 0.05), respectively. Data are presented as mean ± SD from three independent experiments with each running in triplicate. (n = 3, **p < 0.01, ***p < 0.001)

Next, to explore the mechanism through which GKN2 promotes stress-induced apoptosis, we analyzed by western blot the expression of cleaved-poly ADP ribose polymerase (PARP), cleaved-caspase-9, and cleaved-caspase-3 upon GKN2 overexpression or silencing. GKN2 overexpression was associated with increased expression of cleaved-PARP, cleaved-caspase-3 and cleaved-caspase-9 after H_2_O_2_ treatment (Fig. 3a). Contrarily, silencing of *GKN2* decreased the expression of these proteins (Fig. 3b). Pre-treatment with Z-VAD-FMK (VAD) reversed the stress-induced inhibition of MGC and SGC cell proliferation (Additional file 1: Figure S4a).

### Effects of GKN2 on NF-κB and JNK signaling pathways

Next, we sought to investigate the modifications of the intracellular signaling network resulting from H_2_O_2_ treatment by performing a phosphoproteome analysis in *GKN2* silenced cells and control cells: 1318 well-characterized phospho-specific antibodies were included in this array. *GKN2* silenced SGC cells and control SGC cells were treated with H_2_O_2_. A pool of cell lysates including three independent *GKN2* silenced SGC cells at a ratio of 1:1:1 was prepared and equally mixed with the cell lysate of control SGC cells. The oncogenic NF-κB and JNK signaling pathways were selected from the complete data set. Only proteins with changes in their phosphorylation levels of 65% or less and 150% or more upon H_2_O_2_-treatment (and after normalization to untreated control) were considered (Fig. 3c).

We examined the effects of GKN2 on these signaling pathways. We found that NF-κB phosphorylation was reduced in H_2_O_2_-treated MGC and SGC cells overexpressing GKN2 compared with control cells; also, the increased phosphorylation of JNK was observed in cells overexpressing GKN2 after H_2_O_2_ treatment (Fig. 3d). Pre-treatment with bay11–7082 (BAY) promoted stress-induced inhibition of proliferation in MGC and SGC cells (Fig. 3e). Conversely, the phosphorylation of NF-κB was increased and that of JNK was decreased in GKN2-silenced cells (Fig. 3f). Pre-treatment with SP600125 (SP) reversed the stress-induced inhibition of proliferation of MGC and SGC cells (Fig. 3g). Therefore, the activation of JNK and the inactivation of NF-κB are important for GKN2-promoted stress-induced cell death.

### GKN2 sensitizes cell to death through its interaction with Hsc70

**Fig. 4:**
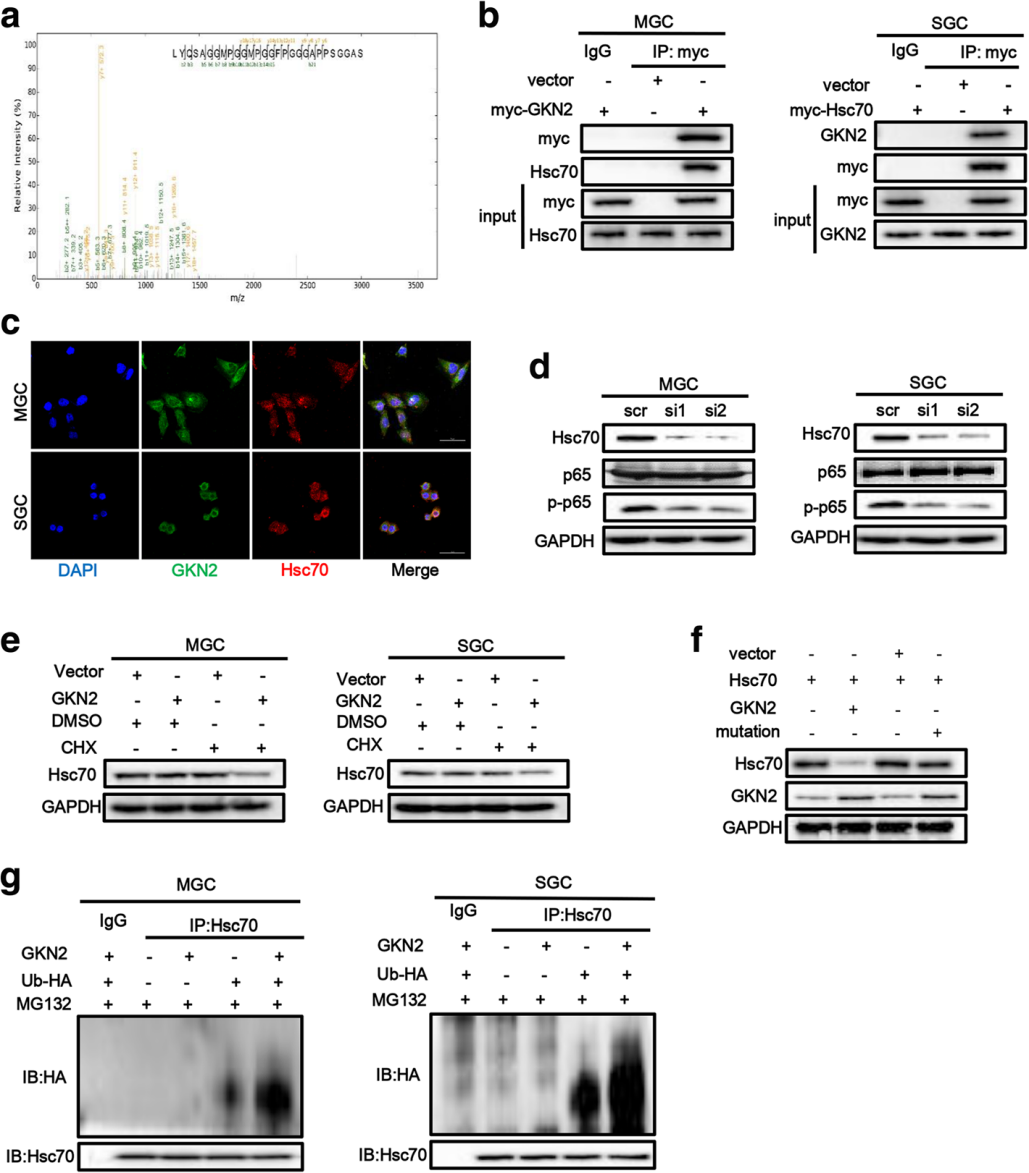
GKN2 directly interacts with Hsc70. **a** Hsc70 was identified by MS as interactor of GKN2. **b** GKN2 interacted with Hsc70 in GC cell lines. MGC cells expressing myc-GKN2 and SGC cells expressing myc-Hsc70 were lysed, subjected to immunoprecipitation using anti-myc antibody, and detected using indicated antibodies. Input: cell lysate without immunoprecipitation. **c** Confocal laser-scanning detected the expression of GKN2 and Hsc70 in GC cell lines. Scale bar: 50 μm. **d** Effect of Hsc70 knockdown. Cells were transfected with siRNA to knockdown Hsc70 and cultured for 48 h. Then indicated proteins were detected. **e** The stability assays of Hsc70 after overexpression of GKN2. The cells were treated with 10 μM cycloheximide (CHX) for 12 h then treated with H_2_O_2_ (300 uM, 6 h) before harvest. **f** The stability assays of Hsc70 after overexpression of GKN2 or its mutation. 293 T cells were transient co-transfected with Hsc70 and GKN2/GKN2 mutation/control plasmid and cultured for 24 h. Then cells were treated with 10 μM cycloheximide for the indicated times then treated with H_2_O_2_ (300 uM, 6 h) before harvest. **g** The expression and ubiquitination of Hsc70 after overexpression of GKN2. Cells were transient transfected with GKN2 and/or HA labeled ubiquitin enzyme (Ub-HA) and cultured for 24 h. With exposure to MG132 (5uM, 2 h), cells were treated with H_2_O_2_ (300 uM, 6 h) before harvest. Then cells were lysed, subjected to immunoprecipitation using anti-Hsc70 antibody and detected using indicated antibodies. MG132: proteasome inhibitor

To further investigate the mechanism of GKN2, we looked for proteins that interact with GKN2. Cell lysates from GKN2-overexpressing and control cells were used for this purpose. We first immunoprecipitated GKN2 (myc-tagged) with an anti-myc monoclonal antibody. The immunocomplexes were then separated by sodium dodecyl sulfate-polyacrylamide gel electrophoresis, followed by band excision and in-gel digestion. Using LC-MS/MS-based quantitative proteomics, we identified more than 19 GKN2-interacting proteins, including Hsc70 (Fig. 4a). Because Hsc70 regulates NF-κB activity, we decided to further investigate the putative interaction between GKN2 and Hsc70. In co-immunoprecipitation assays, we confirmed that GKN2 interacted with Hsc70 (Fig. 4b); specifically, the two proteins co-localized in cytoplasm (Fig. 4c). To further verify the binding specificity, we generated truncated mutants of GKN2 and Hsc70 tagged with myc and HA, and encompassing different domains in the two proteins. Co-immunoprecipitation data demonstrated that the deletion of the BRICHOS domain at the N terminus of GKN2 abrogated the interaction, suggesting that this domain is required for the interaction with Hsc70 (Additional file 1: Figure S5a). Moreover, the nucleotide-binding domain (NBD) of Hsc70 mediated the association with GKN2 (Additional file1: Fig. S5b). These data indicated that Hsc70 was a novel GKN2 interactor in vivo.

**Fig. 5:**
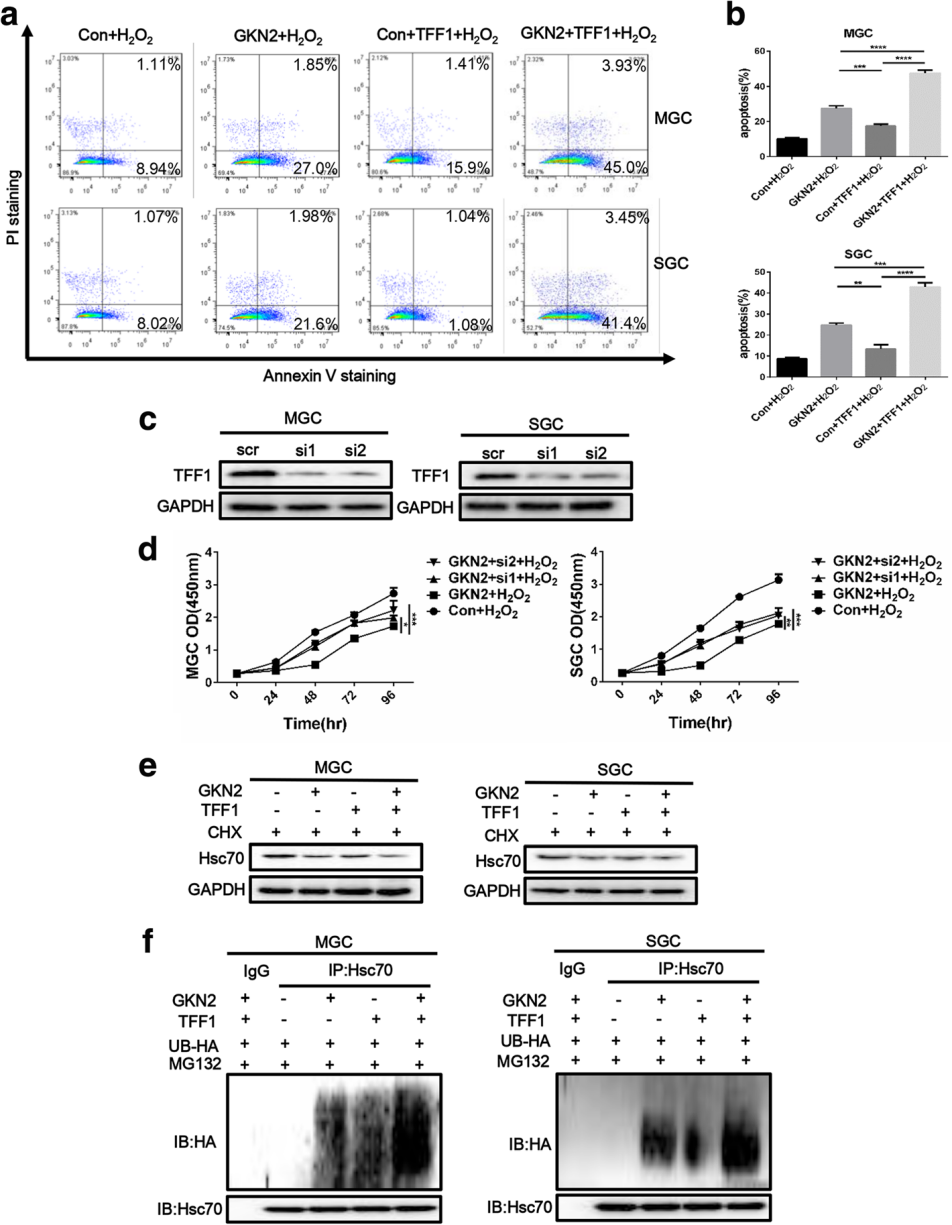
TFF1 enhances the function of GKN2. **a**-**b** Analyses of apoptosis with or without TFF1 after H_2_O_2_ (300 uM, 6 h) treatment. **c** Detection of TFF1 expression after TFF1 was knockdown in GC cell lines. GC cells were transfected with siRNA (100 nM) and 24 h after transfection TFF1 expression was evaluated. **d** Comparison of the proliferation of GC cell lines with or without TFF1 knockdown. Cells were pre-treated with H_2_O_2_ (300 uM) for 6 h. GKN2 overexpressing MGC cells (IC50 528.4 ± 29.5 μM) were significantly more sensitive to H_2_O_2_ compared to GKN2 overexpressing MGC cells with silence of TFF1 (IC50: si1 620.9 ± 33.6, *P* < 0.05; si2 618.4 ± 30.2, *P* < 0.05). In SGC cells, GKN2 overexpressing cells (IC50 539.2 ± 31.6 μM) were significantly more sensitive to H_2_O_2_ compared to GKN2 overexpressing cells with silence of TFF1 (IC50: si1 609.2 ± 23.3, *P* < 0.05; si2 614.1 ± 27.3, *P* < 0.05). **e** The stability assays of Hsc70 after overexpression of TFF1 in control and GKN2-overexpressing GC cell lines. The cells were treated with 10 μM cycloheximide for 12 h then treated with H_2_O_2_ (300uM, 6 h). **f** The expression and ubiquitination of Hsc70 after overexpression of TFF1 in control and GKN2-overexpressing GC cell lines. Cells were treated with H_2_O_2_ (300 uM) for 6 h. Data represent similar results from three independent experiments. (**p < 0.01, ***p < 0.001)

Knockdown of Hsc70 attenuated the effect of GKN2 on p65 phosphorylation in MGC and SGC cells (Fig. 4d) and GKN2 affected Hsc70 stability (Fig. 4e). Furthermore, through Co-IP assay we found Hsc70 interacted with p65 directly (Additional file 1: Figure S5c). Meanwhile, miR-216a further promoted NF-κB pathway by downregulating *GKN2* expression after H_2_O_2_ treatment (Additional file 1: Figure S5d). At same time, we found GKN2 induced the degradation of Hsc70 (Fig. 4e) and promoted ubiquitinoylation of Hsc70 in GC cells after H_2_O_2_ treatment through the detection of indicated proteins (Fig. 4g). However, the natural mutation of GKN2 could not facilitate the degradation of Hsc70 in 293 T cells were co-transfected with Hsc70 vector and GKN2 mutation vector then treated with H_2_O_2_ (Fig. 4f). The expression of GKN2 may suppress cancer cell proliferation through a TFF1-dependent manner. To investigate if the oxidative stress-induced anti-proliferative and pro-apoptotic effects of GKN2 in gastric cancer cell were TFF1-dependent, we overexpressed or knocked down *TFF1* in GKN2-overexpressing and control cells. We found that synchronous overexpression of GKN2 and TFF1 promoted apoptosis of cells compared with other groups (Fig. 5a, b). Upon knockdown of *TFF1* (Fig. 5c), CCK8 assays revealed that the overexpression of GKN2 in MGC and SGC cells still inhibited cell proliferation (Fig. 5d). Meanwhile, TFF1 augmented the effect of GKN2 on Hsc70 degradation (Fig. 5e) and promoted ubiquitinoylation (Fig. 5f). Taken together, these results demonstrated that the anti-proliferative and pro-apoptotic effects of GKN2 were TFF1-independent, although TFF1 could contribute to the effects of GKN2.

### GKN2 promotes the therapeutic effects of piperlongumine (PL) in a xenograft mouse model

**Fig. 6:**
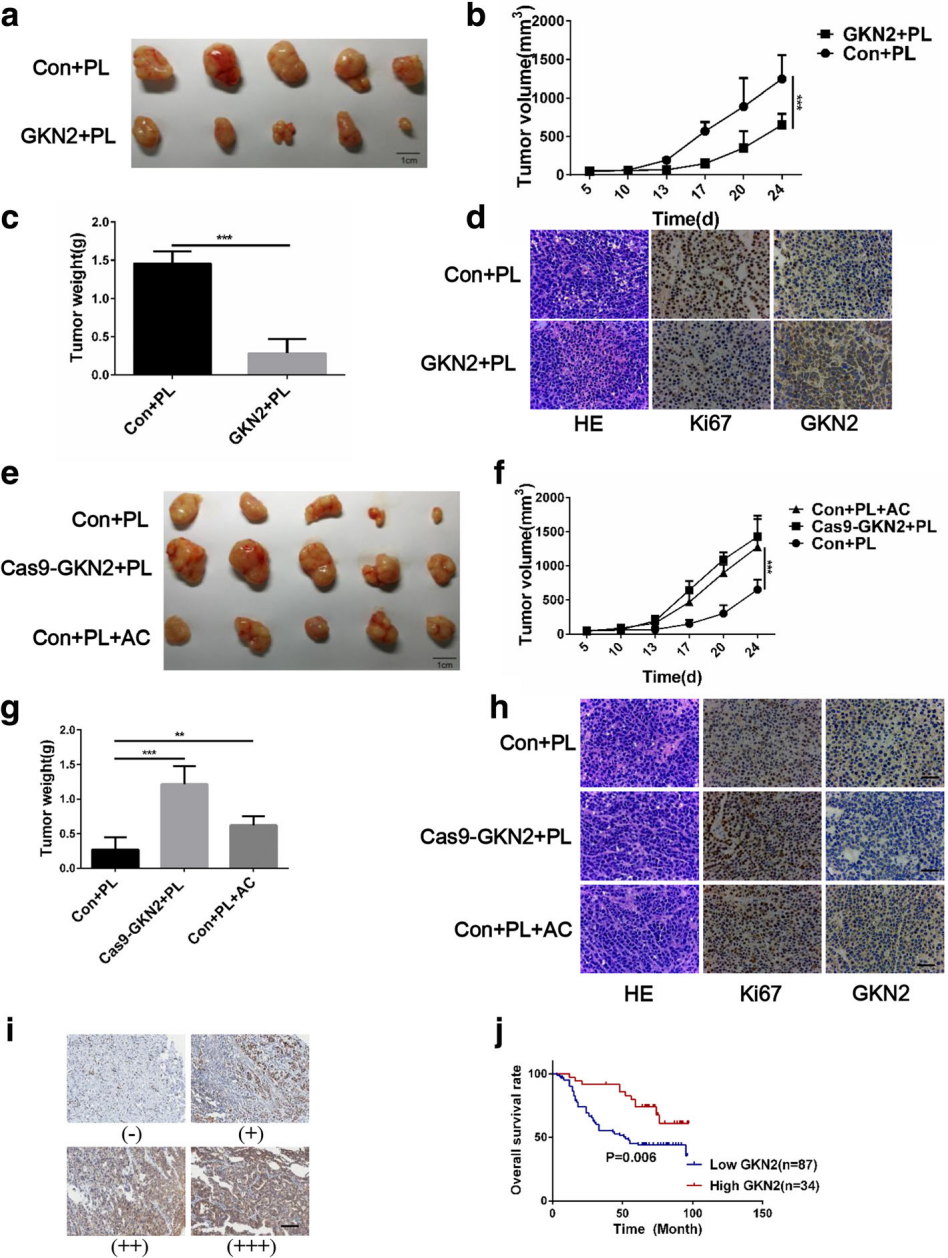
GKN2 represses the tumorigenicity of GC cells under stress condition. **a** Forced expression of GKN2 suppresses the tumorigenicity of MGC in nude mice with PL treatment (*n* = 5). **b**-**c** Tumor volume and weights of xenografts tumors of MGC-con and MGC-GKN2 are significantly different. **d** Representative images of staining of HE, GKN2 and Ki67 in xenograft tumors of MGC-con and MGC-GKN2. Scale bar: 200 μm. **e** Silencing of GKN2 enhances the tumorigenicity of SGC in nude mice (*n* = 5). Mice were intraperitoneal injected with acetylcysteine (AC, 300 mg/kg) 2 h before administration of PL. (**f**-**g**) Tumor volume and weight of xenografts tumors of SGC-con and SGC-cas9-GKN2 were significantly different. Complementation of AC enhanced effect of the tumorigenicity in vivo. (**h**) Representative images of staining of HE, GKN2 and Ki67 in xenograft tumors. Scale bar: 200 μm. (**i**) IHC staining of human GC tissues using GKN2-specific antibody; classification of samples according to the intensity of staining of GKN2 expression. Scale bar: 200 μm. **j** Kaplan-Meier curves for GC patients’ overall survival in the patients with high GKN2 expression and low GKN2 expression (*n* = 121, *p* = 0.006). The endpoint event was defined as mortality **p < 0.01, ***p < 0.001

PL, a natural product isolated from the long pepper *Piper longum L*, is selectively toxic to cancer cells in vitro and in vivo [24] and PL treatment increases ROS levels in cancer cells. To verify the function of GKN2 in vivo, MGC cells overexpressing GKN2 and SGC silenced for *GKN2*, or control MGC and SGC cells were subcutaneously injected in mice. Four weeks after injection of PL, the mice were sacrificed and the tumors taken out and weighed. The mean tumor weight and volume were significantly lower in the GKN2-overexpressing group than in the control group (overexpressing the empty vector; Fig. 6a-c). On the other hand, silencing of *GKN2* in SGC cells resulted in the development of larger tumors compared with the control cells and the effects of PL treatment was attenuated by administration of acetylcysteine (AC) (Fig. 6e-g). Consistently, the expression of GKN2 in the xenograft tumors was negatively correlated with Ki67, a cellular marker for proliferation (Fig. 6d, h). These data, together with the findings reported above, suggested that GKN2 repressed GC cells proliferation both in vitro and in vivo.

### Prognostic significance of GKN2 in patients with GC

It has been reported that oxaliplatin increased generation of ROS, ultimately leading to the activation of apoptotic pathways [26]. To further evaluate the clinical significance of GKN2 in GC, we determined GKN2 levels in 121 patients with GC and treated with oxaliplatin after surgery (Fig. 6i). Importantly, the patients with higher GKN2 expression levels (++ and +++) had better overall survival than those with lower GKN2 expression levels (− and +; Fig. 6j). Univariate analyses using the Cox hazard regression model identified GKN2 expression, stage and metastasis as prognostic indicators of overall survival for patients with GC (Table 1). Multivariate analysis further demonstrated the significant contribution of higher GKN2 expression to better outcomes of the patients (hazard ratio, 0.464; 95% CI, 0.239–0.901; *P* = 0.023; Table 1) and indicated that GKN2 was an independent and significant prognostic factor for patients with GC treated with oxaliplatin.

**Table 1:** Analyses of time to disease progression in advance GC patients under the treatment of oxaliplatin

Factors	N	Univariate analysis		Multivariate analysis	
		HR (95% CI)	P	HR (95% CI)	P
Gender					
Female	34	1.140 (0.643–2.022)	0.653		
Male	87				
Age					
< 60	51	1.345 (0.799–2.264)	0.265		
≥ 60	70				
Stage					
I & II	61	2.776 (1.627–4.734)	< 0.001	2.106 (1.221–3.631)	0.007
III & IV	60				
Metastasis					
No	45	3.978 (2.062–7.675)	< 0.001	3.534 (1.818–6.868)	< 0.001
Yes	76				
Type					
Diffuse	105	0.658 (0.283–1.571)	0.331		
Intestinal	16				
GKN2					
Low (− & +)	87	0.544 (0.215–0.798)	0.008	0.464 (0.239–0.901)	0.023
High (++&+++)	34				

Staining intensity, negative: -, weak positive: +, moderate positive: ++, strong positive: +++

## Discussion

In this study, we addressed the molecular mechanism underlying the tumor suppressor function of GKN2 in GC. GKN2 significantly reduced proliferation and promoted apoptosis of GC cells under stress. Overexpression of GKN2 and treatment with H_2_O_2_ synergistically inhibited cell proliferation and promoted apoptosis by upregulating cleaved (active) caspase-3 and active caspase-9, important effectors of apoptosis. Interestingly, H_2_O_2_ induced the upregulation of GKN2 in GC cells. These findings are consistent with previous reports and strongly suggest that GKN2 functions as a tumor suppressor by regulating a set of proteins involved in apoptosis.

The expression of GKN2 decreases both in GC tissues and cell lines [27, 28], so we set out to determine whether this process was epigenetically regulated. However, we did not find any CpG island in the promoter region of *GKN2*. Interestingly, we found that miR-216a, which is upregulated in GC, could potentially inhibit *GKN2* expression by binding to a plausible recognition site in the 3’-UTR of *GKN2*. Luciferase assays confirmed that miR-216a regulated the expression of GKN2. Additionally, miR-216a mimics suppressed the expression of *GKN2* and *GKN2* silencing in GC cells was reversed by miR-216a inhibitors. Furthermore, GKN2 expression was inversely correlated with that of miR-216a in GC tissues and cell lines.

Overexpression of GKN2 and treatment with H_2_O_2_ synergistically promoted cell apoptosis by inhibiting NF-κB and activating JNK. The inactivation of NF-κB by GKN2 has previously been reported in SGC cells. In this study, we have uncovered a new route for the inhibition of NF-κB pathway, mediated by GKN2 during oxidative stress. In cell based assays, we found that, after treatment with H_2_O_2_, GKN2 suppressed phosphorylation of NF-κB and the activation of this pathway. The effects of ROS, induced by H_2_O_2_, were largely indirect; however, our finding suggested the mechanism behind changes induced by GKN2 is stress-related.

*GKN2*, a stomach-specific gene, has been researched upon for more than 10 years; recently, it has been proposed it acts as a tumor suppressor in many human cancer cell lines. The heterodimeric interaction between GKN2 and TFF1 played an important in inhibition of GC [29]; thereafter, its anti-proliferative and pro-apoptotic effects on GC cells were further substantiated and other mechanisms of GKN2 action emerged [30], including the recent indication that GKN2 functions as a GKN1 inhibitor, contributing to the homeostasis of gastric mucosa. GKN2 also play a significant role in regulation of gastric inflammation [31].

To identify the molecular details of GKN2 action on NF-κB and JNK, we investigated the protein(s), which directly interact with GKN2, by co-immunoprecipitation followed by MS. We found that GKN2 bound Hsc70, which is important for NF-κB transcriptional activity, and promoted its degradation by ubiquitination. Several studies indicate that GKN2 forms a dimer with TFF1 through two heterologous disulfide bonds, and GKN2 and TFF1 show a strong correlation in clinical samples and related cell lines. A recent study has found that GKN2 could up-regulate levels of TFF1 in GKN2-overexpressing GC. However, we found that the anti-proliferative and pro-apoptotic effects of GKN2 were TFF1-independent. Therefore, although the findings presented in our study are in agreement with many of the experimental results presented in previous studies, our study comes to different conclusions regarding the mechanisms through which GKN2 mediates its effect.

ROS are toxic at certain levels, due to their reaction with proteins, lipids, and nucleic acids. The correct cellular response to ROS production is therefore critical in preventing further oxidative damage. ROS can induce both apoptotic and necrotic cell death depending on the severity of the oxidative stress damage [32–34] Genes targeted by canonical NF-κB promote cellular survival. Therefore, it is not surprising that ROS can modulate an NF-κB response and, on the other hand, ROS-induced damage is attenuated by NF-κB target genes.

The crosstalk between the NF-κB-induced cell survival pathway and the JNK-induced cell death pathway is affected by ROS production [35–37] and occurs in multiple ways [38–42]. This crosstalk is known to prevent sustained JNK activation and massive cell death through apoptosis and necrosis [36]. There is a positive feedback loop between ROS-dependent JNK activation and the generation of JNK/SAB-dependent mitochondrial ROS [43, 44]. NF-κB signaling induces the transcription of both anti-apoptotic genes and genes involved in the downregulation of intracellular ROS levels. The production of antioxidants resulting from NF-κB activation plays a vital role in balancing ROS effects. Two major players in this context are manganese superoxide dismutase-2 and catalase, both of which counteract apoptosis by neutralizing mitochondrial ROS [45, 46]. On the other hand, ROS contribute to apoptosis by inducing mitochondrial outer-membrane permeabilization and JNK activation. The mitochondrial effect on apoptosis is largely mediated by proteins of the B-cell lymphoma 2 (Bcl2) family, such as Puma and Bim [47, 48]. As a result, caspase-9 and caspase-3 are activated and induce apoptosis. However, the pro-apoptotic activity of these molecules can be attenuated by the anti-apoptotic proteins B-cell lymphoma-extra large (Bcl-XL) as well as Bcl2, whose expression is regulated by NF-κB [49].

## Conclusions

In summary, we demonstrated GKN2 might increase sensitivity of GC cells to the drugs which increase ROS levels in tumors. Inhibition of the interaction between GKN2 and Hsc70 could attenuate the effects induced by GKN2. TFF1 could promote the function of GKN2, however, the anti-proliferative and pro-apoptotic effects of GKN2 were TFF1-independent. GKN2 overexpression could be used to determine the subgroup of patients to obtain the more favorable outcome of oxaliplatin treatment and might be used as biomarker of the prognosis of this cancer.

## Additional files

Additional file 1: **Figure S1.** Expression of GKN2 in GC cell lines. **Figure S2.** GKN2 silencing suppresses the sensitivity of GC cell lines to H2O2. **Figure S3.** H2O2 induces ROS-dependent mitochondrial dysfunction. **Figure S4.** GKN2 inhibits proliferation via caspase pathway. **Figure S5.** GKN2 interacts with Hsc70 and regulates NF-κb pathway through Hsc70 (DOCX 2348 kb)

Additional file 2: **Table S1.** Clinicopathological characteristics of GC patients for analyzing the clinical significance of mir-216a expression (*n* = 35). (DOCX 14 kb)

## Data Availability

The datasets supporting the conclusions of this article are included within the article and its additional files.

## Abbreviations

AC: acetylcysteine
BAY: Bay11–7082
CCK8: Cell counting kit-8
CHX: Cycloheximide
DCFH-DA: Redox-sensitive fluorescent probe 2’-,7’-dichlorofluorescein diacetate
GC: Gastric cancer
GDDR: Gastric dramatic down-related gene
GKN2: Gastrokine 2
GSH: Glutathione
H_2_O_2_: Hydrogen peroxide
Hsc70: Heat shock cognate 71 kDa protein
JNK: c-Jun N-terminal kinase
MGC: MGC-803
miRNA: MicroRNA
MS: Mass spectrometry
NBD: Nucleotide-binding domain
NF-κB: Nuclear factor kappa-light-chain-enhancer of activated B cells
PBS: Phosphate-buffered saline
PL: Piperlongumine
qRT-PCR: Quantitative real-time polymerase chain reaction
ROS: Oxygen species
SD: mean ± standard
SGC: SGC-7901
siRNA: Small interfering RNA
SP: SP600125
TFF: Trefoil factor
VAD: Z-VAD-FMK
