# Supplementary material for: GKN2 promotes oxidative stress-induced gastric cancer cell apoptosis via the Hsc70 pathway
Source: J Exp Clin Cancer Res. 2019 Aug 5;38:338. doi: 10.1186/s13046-019-1336-3 (PMC6683576; doi:10.1186/s13046-019-1336-3)
Supplement: Supplementary file 1 — Figure S1. Expression of GKN2 in GC cell lines. Figure S2. GKN2 silencing suppresses the sensitivity of GC cell lines to H2O2. Figure S3. H2O2 induces ROS-dependent mitochondrial dysfunction. Figure S4. GKN2 inhibits proliferation via caspase pathway. Figure S5. GKN2 interacts with Hsc70 and regulates NF-κb pathway through Hsc70 (DOCX 2348 kb) [file 13046_2019_1336_MOESM1_ESM.docx]

**FigureS1**

**
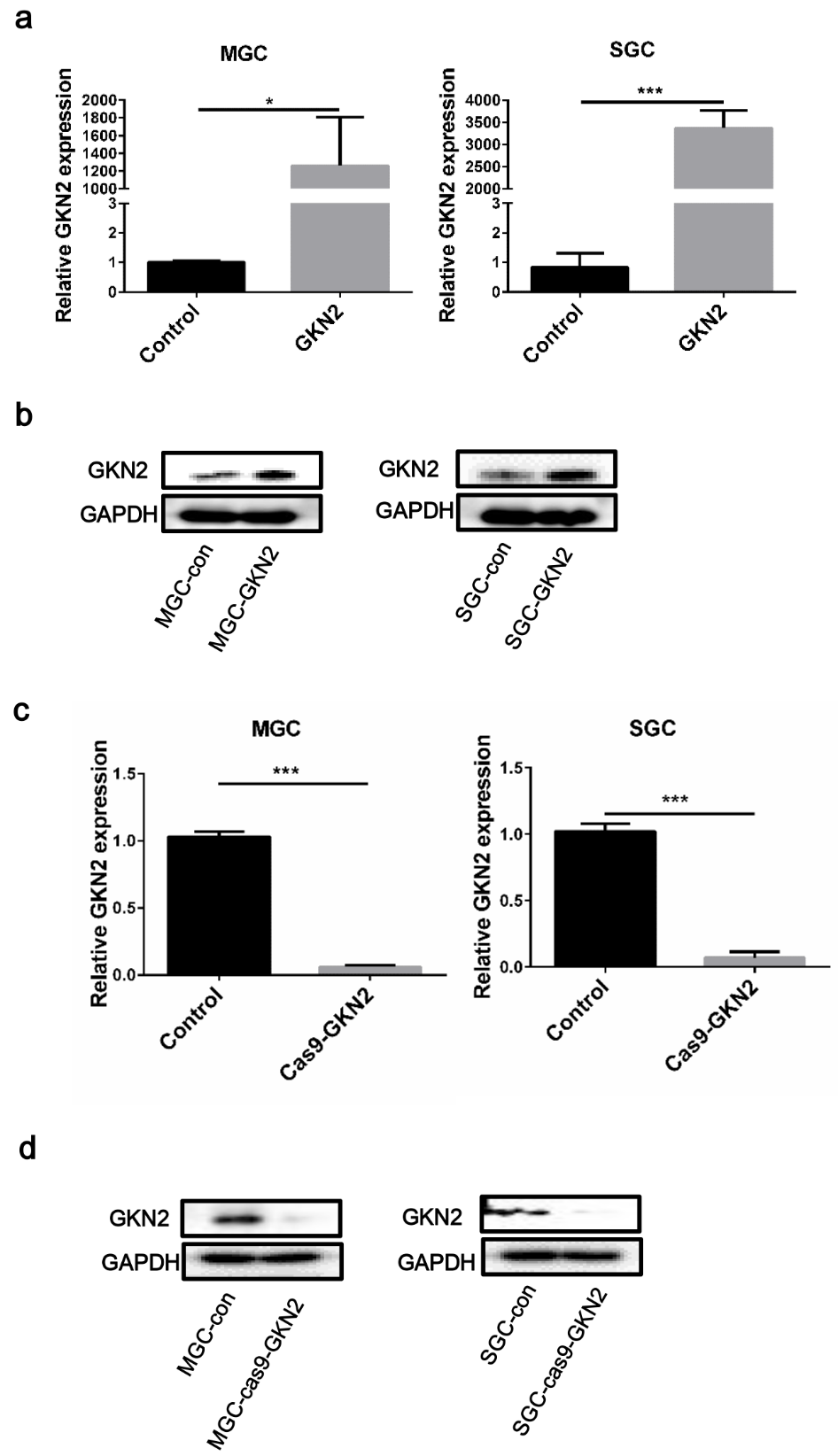
**

**Figure S1**. Expression of GKN2 in GC cell lines. (a-b) The overexpression of GKN2 was achieved in MGC and SGC cells by stable transfection. Expression of GKN2 was measured with qRT-PCR and western blot. (c-d) The silencing of GKN2 was achieved in MGC and SGC cells by CRISPR/Cas9. Expression of GKN2 was measured with qRT-PCR and western blot. (western blot and qRT-PCR repeated in three independent experiments). Columns are the mean values of triplicates; the error bar indicates the SEM. Asterisks indicate statistically significant differences from each other; *p < 0.05, ***p < 0.001.

**FigureS2**

**
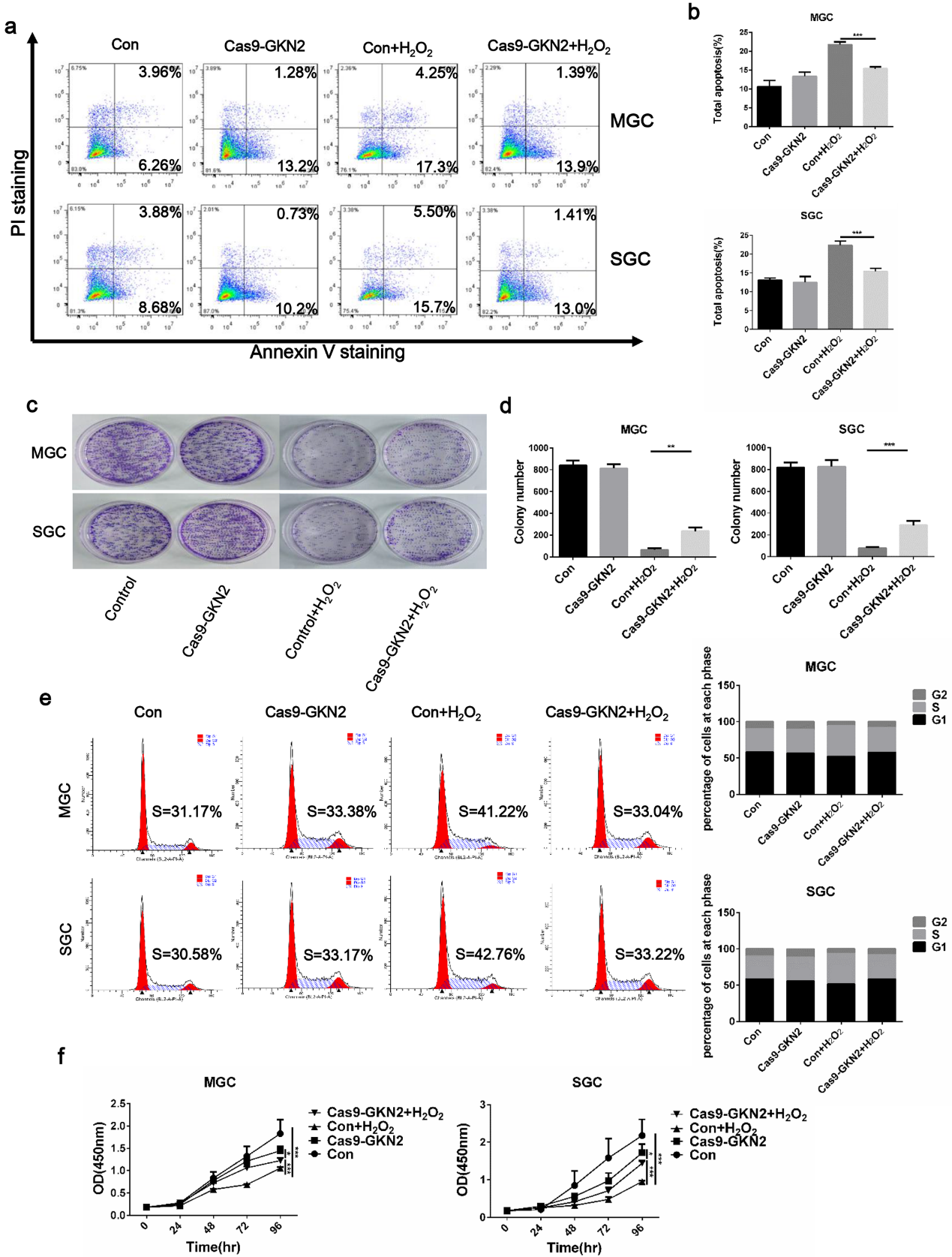
**

**Figure S2.** GKN2 silencing suppresses the sensitivity of GC cell lines to H_2_O_2_. (a-b) Analyses of apoptosis with or without H_2_O_2_ (300 uM) concentration for 6h in GC cell lines. (c-d) Comparison of colony formation in MGC and SGC cells with or without H_2_O_2_ (300 uM, 6h) pre-treatment. (e) Analyses of cell cycle after PI staining in GC cell lines after H_2_O_2_ (300 uM, 6h) treatment. Data are presented as the mean of three independent experiments. (f) Comparison of proliferation in MGC and SGC cells with or without H_2_O_2_ (300uM, 6h) pre-treatment. GKN2 silencing MGC cells (IC50 807.4±45.4 μM) and SGC cells (IC50 836.8±30.5 μM) were significantly less sensitive to H_2_O_2_ compared to control MGC cells (IC50 660.4±29.4 μM, P < 0.01) and SGC cells (IC50 663.0±25.6 μM, P < 0.01), respectively. Data are presented as mean ± SD from three independent experiments with each running in triplicate. (n = 3, *p < 0.05, **p < 0.01, ***p < 0.001).

**FigureS3**

**
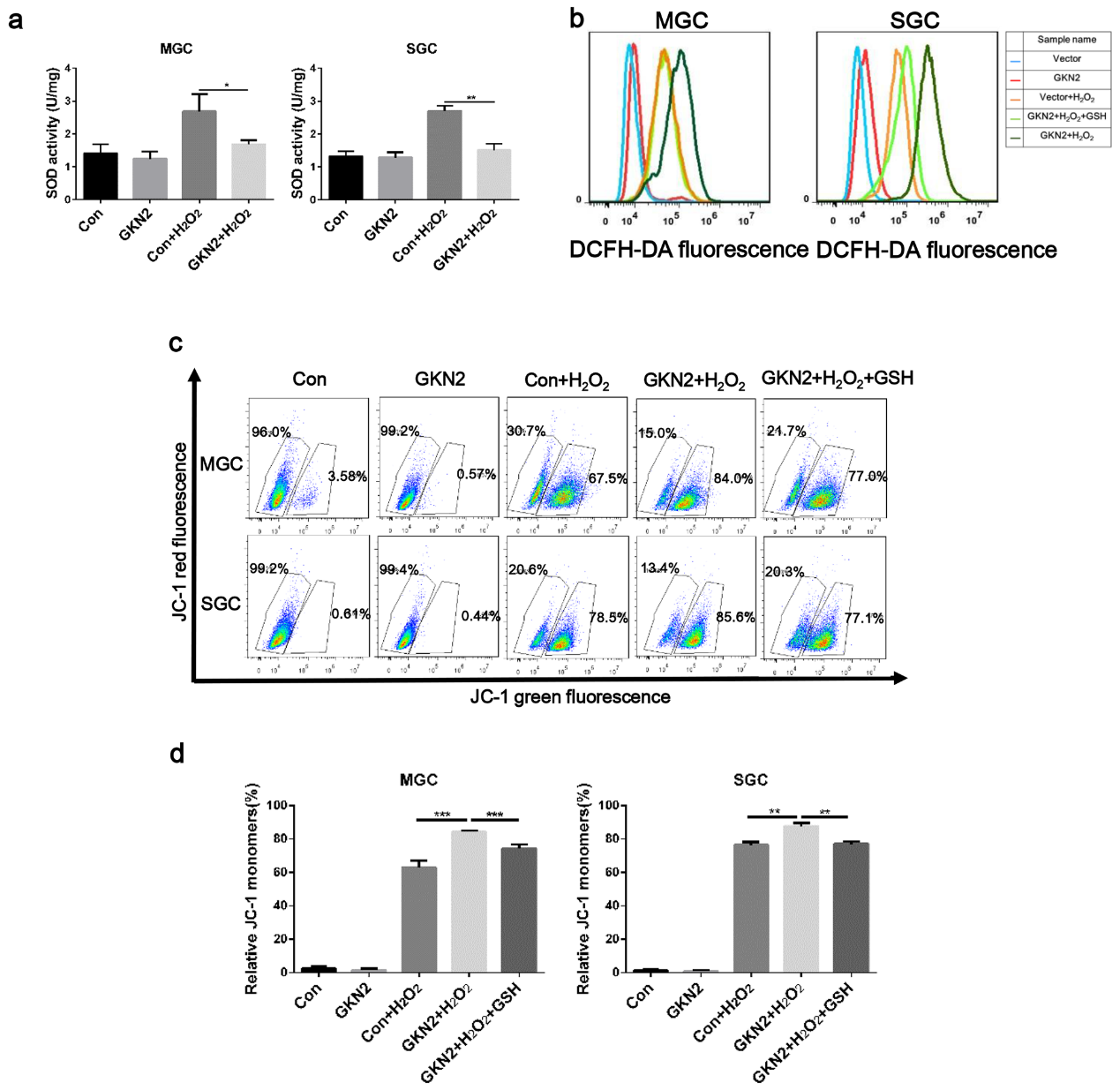
**

**Figure S3.** H_2_O_2_ induces ROS-dependent mitochondrial dysfunction. (a) GKN2 inhibited H_2_O_2_-induced increase of SOD activity. (b) Intracellular ROS generation induced by H_2_O_2_ was measured in MGC and SGC cells by staining with DCFH-DA and flow cytometry analysis. Cells were incubated with H_2_O_2_ (300 uM) for 6h. Cells were incubated with GSH (2mM, 2h) before H_2_O_2_ treatment. Intracellular ROS generation was measured by flow cytometry. (c-d) Treatment of cells with H_2_O_2_ obviously decreased the mitochondrial membrane potential (△ψm) in cells over-expressing GKN2 after treatment with H_2_O_2_ for 6h were stained with JC-1. (*p <0.05, **p <0.01, ***p <0.001).

**FiugreS4**

**
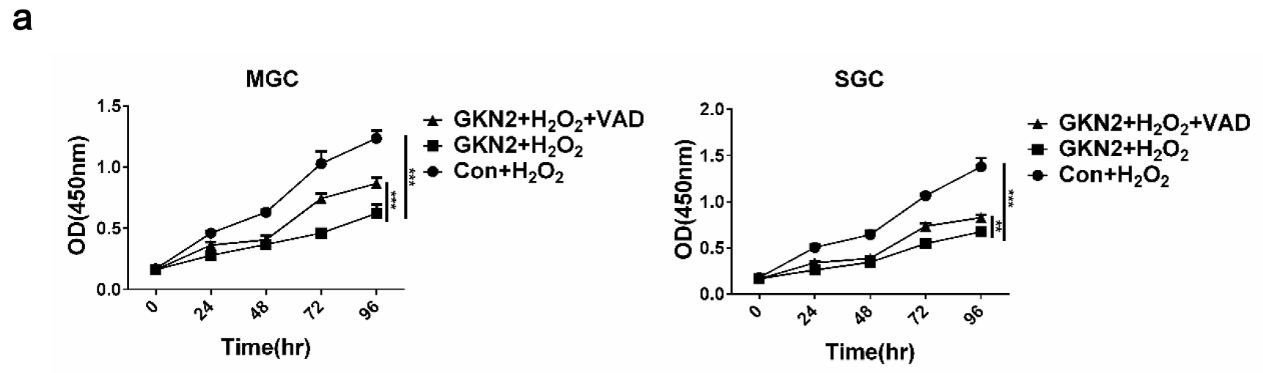
**

**Figure S4.** GKN2 inhibits proliferation via caspase pathway. (a) Comparison of the proliferation of GC cell lines after H_2_O_2_ (300 uM) treatment for 6h with or without inhibition of caspase by 50 uM VAD. Cells were pretreated with the inhibitor for 2h and maintained in culture. GKN2 overexpressing MGC cells (IC50 742.6±34.1 μM) and SGC cells (IC50 722.6±21.4 μM) with inhibition of caspase were significantly less sensitive to H_2_O_2_ compared to control MGC cells (IC50 660.4±29.4 μM, P < 0.05) and SGC cells (IC50 663.0±25.6 μM, P < 0.05), respectively. Data are presented as mean ± SD from three independent experiments with each running in triplicate. (n = 3, *p <0.05, **p <0.01, ***p <0.001).

**FigureS5**

**
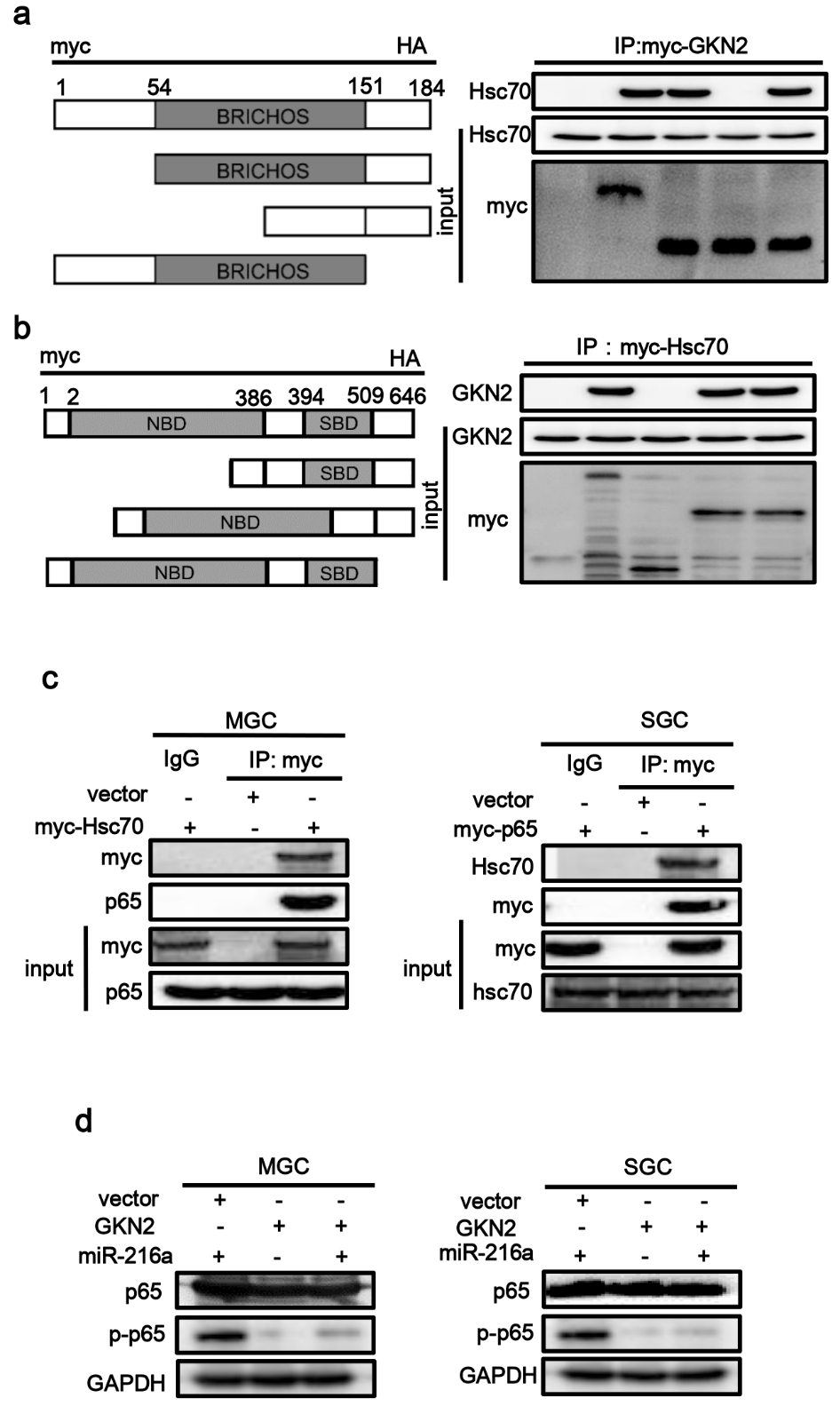
**

**Figure S5.** GKN2 interacts with Hsc70 and regulates NF-κb pathway through Hsc70. (a) BRICHOS domain of GKN2 was required for the interaction of GKN2 and Hsc70. 293T cells were co-transfected with myc-GKN2 and indicated truncates for 24 hours. Cells were then harvested for co-IP assays and detected using anti-myc antibody. input: cell lysate without immunoprecipitation. (b) The NBD domain of Hsc70 mediated the binding to GKN2. 293T cells were co-transfected with myc-Hsc70 and indicated truncates for 24 hours. Cells were then harvested for co-IP assays and detected using anti-myc antibody. input: cell lysate without immunoprecipitation. (c) Hsc70 interacted with p65 in GC cell lines. MGC cells expressing myc-Hsc70 and SGC cells expressing myc-p65 were lysed, subjected to immunoprecipitation using anti-myc antibody, and detected using indicated antibodies. input: cell lysate without immunoprecipitation. (d) Detection of protein expression in cells transfected with mir-216a then treated with H_2_O_2_ (300 uM) for 6h.
