# Supplementary material for: GKN2 promotes oxidative stress-induced gastric cancer cell apoptosis via the Hsc70 pathway
Source: J Exp Clin Cancer Res. 2019 Aug 5;38:338. doi: 10.1186/s13046-019-1336-3 (PMC6683576; doi:10.1186/s13046-019-1336-3)
Supplement: Supplementary file 2 — Table S1. Clinicopathological characteristics of GC patients for analyzing the clinical significance of mir-216a expression (n = 35). (DOCX 14 kb) [file 13046_2019_1336_MOESM2_ESM.docx]

**Table S1: Clinicopathological characteristics of GC patients for analyzing mir-216a expression (n=35)**

| Characteristics | Case number | percentage |
| --- | --- | --- |
| Sex |  |  |
| Female | 14 | 40.0% |
| Male | 21 | 60.0% |
| Age (years) |  |  |
| <60 | 11 | 31.4% |
| ≥60 | 24 | 68.6% |
| Stage |  |  |
| Ⅰ & Ⅱ | 16 | 45.7% |
| Ⅲ & Ⅳ | 19 | 54.3% |
| Lymph node |  |  |
| negative | 12 | 34.3% |
| positive | 23 | 65.7% |
| Type |  |  |
| diffuse | 15 | 42.9% |
| intestinal | 20 | 57.1% |
